# Acceptance and Commitment Therapy Preceded by Attention Bias Modification on Residual Symptoms in Depression: A 12-Month Follow-Up

**DOI:** 10.3389/fpsyg.2019.01995

**Published:** 2019-08-29

**Authors:** Tom Østergaard, Tobias Lundgren, Ingvar Rosendahl, Robert D. Zettle, Rune Jonassen, Catherine J. Harmer, Tore C. Stiles, Nils Inge Landrø, Vegard Øksendal Haaland

**Affiliations:** ^1^Department of Psychiatry, Sørlandet Hospital, Arendal, Norway; ^2^Department of Psychology, Clinical Neuroscience Research Group, University of Oslo, Oslo, Norway; ^3^Department of Clinical Neuroscience, Center for Psychiatry Research, Karolinska Institutet and Stockholm Health Care Services, Stockholm County Council, Stockholm, Sweden; ^4^Department of Psychology, Wichita State University, Wichita, KS, United States; ^5^Psychopharmacology and Emotion Research Laboratory, University Department of Psychiatry, Oxford, United Kingdom; ^6^Department of Psychology, Norwegian University of Science and Technology, Trondheim, Norway; ^7^Division of Psychiatry, Diakonhjemmet Hospital, Oslo, Norway

**Keywords:** acceptance and commitment therapy, attentional bias modification, combined treatment, depression, residual symptoms

## Abstract

Depression is a highly recurrent disorder with limited treatment alternatives for reducing risk of subsequent episodes. Acceptance and commitment therapy (ACT) and attention bias modification (ABM) separately have shown some promise in reducing depressive symptoms. This study investigates (a) if group-based ACT had a greater impact in reducing residual symptoms of depression over a 12-month follow-up than a control condition, and (b) if preceding ACT with ABM produced added benefits. This multisite study consisted of two phases. In phase 1, participants with a history of depression, currently in remission (*N* = 244), were randomized to either receive 14 days of ABM or a control condition. In phase 2, a quasi- experimental design was adopted, and only phase-1 participants from the Sørlandet site (*N* = 124) next received an 8-week group-based ACT intervention. Self-reported and clinician-rated depression symptoms were assessed at baseline, immediately after phase 1 and at 1, 2, 6, and 12 months after the conclusion of phase 1. At 12-month follow-up, participants who received ACT exhibited fewer self-reported and clinician-rated depressive symptoms. There were no significant differences between ACT groups preceded by ABM or a control condition. There were no significant differences between ACT groups preceded by ABM or a control condition. Group-based ACT successfully decreased residual symptoms in depression over 12 months, suggesting some promise in preventing relapse.

## Introduction

Because a large portion of those diagnosed with major depressive disorder (MDD) relapse after initially having recovered (Solomon et al., 2000), secondary prevention is critical in long-term management of the disorder (National Institute for Health and Clinical Excellence, 2009). The benefit depressed clients receive from pharmacological and/or psychological interventions is limited (Turner et al., 2008; Cuijpers et al., 2010; Bockting et al., 2018), with one study finding that only 58% responded to treatment (DeRubeis et al., 2005). The individual and societal costs of recurrent depression episodes are considerable, which underline the importance of further investigating and developing novel interventions (Smit et al., 2006). A step in this direction is to investigate combinations of treatments that reflect a comprehensive understanding of the disorder from multiple perspectives (Beck and Bredemeier, 2016) in considering an array of specific factors that increase risk of repeated episodes of depression (Southwick et al., 2005).

Consistent with a cognitive model (Beck, 2008), research suggests that biased attention toward negative information may play an important role in the development and maintenance of depression (Gotlib et al., 2004; De Raedt and Koster, 2010). Evidence of an attentional bias toward negative stimuli has been reported in both previously (Joormann and Gotlib, 2007) and currently depressed individuals (Gotlib et al., 2004), and in never-depressed individuals at high risk because of familial history (Joormann et al., 2007). This suggests that attentional bias may constitute an important vulnerability factor for depression and therapeutic target, rather than a simple marker of lowered mood. Ostensibly, the most direct strategy for reducing such bias would be through approaches that specifically focus on cognitive processing such as attention bias modification (ABM) (Koster et al., 2011).

Attention bias modification is a simple computerized technique that seeks to shift attention toward more positive stimuli. In this procedure, participants are instructed to respond to a probe placed either behind positive, negative, or neutral stimuli. The probe is placed mainly behind positive stimuli to develop a habit of automatically directing attention toward such stimuli, thus establishing a positive attentional bias. By modifying attentional biases, ABM seeks to change automated and implicit attentive processes and promote more adaptive emotion regulation necessary to prevent of depression (Joormann and Gotlib, 2007; LeMoult and Gotlib, 2018).

Some studies have found ABM to reduce residual symptoms in participants in remission from depression (Wells and Beevers, 2010; Browning et al., 2012; Beevers et al., 2015; Yang et al., 2015). However, overall findings of its efficacy have been mixed, and definitive conclusions in a number of studies have been limited by small sample sizes and poor trial methodology (Hallion and Ruscio, 2011; Cristea et al., 2015; Jones and Sharpe, 2017). A recent large study by Jonassen et al. (2019) found that 2 weeks of ABM produced a small, but statistically significant improvement in clinician-ratings of residual symptoms of depression. Consistent with cognitive models of emotional disorders, the degree of symptom improvement increased with relatively more positive attentional bias within the ABM group.

There also has been increasing interest in targeting attentional bias through further means, such as combining psychotherapeutic approaches with those focusing on cognitive processing. Koster and Bernstein (2015) suggested that combining ABM and psychotherapy might be more beneficial than “stand-alone” treatment, and a recent study by Lazarov et al. (2018) found that ABM augmented cognitive behavioral group therapy for social anxiety. Attentional bias in depressed individuals in particular has also been addressed and challenged, albeit perhaps differently than in ABM, by several cognitive-behavioral approaches such as metacognitive therapy (Wells, 2011) and acceptance and commitment therapy (ACT) (Hayes et al., 2012). A recent study by Vazquez et al. (2018) found that cognitive behavioral therapy significantly contributed to a change in attention bias in depressed participants, thus providing preliminary evidence of the effect of psychotherapy on attention bias.

As its name suggests, ACT combines acceptance and mindfulness processes with commitment and behavior change processes (Hayes et al., 2012). ACT regards depression as a secondary emotion that emerges as a result of unsuccessful efforts to control normal and adaptive affective reactions; such as sadness, a sense of loss, and disappointment; to distressing life events (Zettle, 2015). Ruminative brooding over negative emotional states and related thoughts, in particular, has been shown to play a central role in the initiation, maintenance and recurrence of depression (Zettle and Hayes, 2002; Treynor et al., 2003; Bagby et al., 2004; Nolen-Hoeksema et al., 2008). In ACT, clients as an alternative are taught and encouraged to attend to such unwanted psychological events with mindful acceptance (Levin et al., 2015). Doing so seeks to change the function of such internal experiences (i.e., how one relates to sad thoughts and feelings), and thereby supports increased attentional flexibility along with a more compassionate and accepting attitude toward unwanted private events (Hayes et al., 2011).

Acceptance and commitment therapy has been found to be an efficacious treatment for depression when evaluated in individual, self-help, and group formats (Forman et al., 2007; Fledderus et al., 2012; Folke et al., 2012; Society of Clinical Psychology, 2016; A-Tjak et al., 2018; Kyllönen et al., 2018). A study by Bohlmeijer et al. (2011) investigating ACT as an early group intervention for the general public found that it significantly reduced self-reported symptoms of depression in a 3-month follow-up. Insofar as residual depressive symptoms have been identified as one of the strongest predictors for episodic relapse (Paykel, 2008), these results suggest that ACT might be helpful in secondary prevention of depression. However, the lack of diagnostic assessment and short follow-up in their study limit the findings. Furthermore, the preventive effect of ACT on recurrent depression remains unclear as no studies have been undertaken with this population. Further studies are needed to investigate the impact of ACT on residual symptoms of depression in a more extensive follow-up.

Although ABM and ACT appear to be quite different approaches, they may complement each other to the degree that both target attention. ACT, in particular, may primarily complement ABM through its emphasis on mindfulness. In short, individuals who receive both ABM and ACT may be less likely to (a) even be aware of stimuli and events that might otherwise trigger rumination, and (b) to be more accepting of that which is still explicitly noticed. ABM and ACT might also complement each other through different levels of cognitive processing. ABM involves “lower-order” cognitive processes incorporating automatic and implicit attention without the involvement of apparent language or culturally based processes. ACT, on the other hand, implicates more “higher-order” cognitive processes in targeting attention more overtly and explicitly.

Our study protocol article (Østergaard et al., 2018) offers a more detailed account on how ABM and ACT may complement each other. In order to maximize the potential augmenting effect of ABM and not overwhelm and confuse participants, we chose to conduct 2 weeks of ABM training first, immediately followed by 8 weeks of group-based ACT. This design enabled us to address two fundamental questions. First, we wanted to investigate if group-based ACT had a greater impact in reducing residual symptoms of depression than a control condition. Second, we wanted to explore if participants who sequentially received ABM and ACT experienced greater benefits than those who only received ACT.

## Materials and Methods

### Participants

The current preregistered trial (NCT02648165) was an extension of a larger double-blinded randomized trial (RCT) (NCT02658682) that investigated the direct effects of ABM training. In this multisite study, participants from Oslo and Sørlandet (The south of Norway) were recruited from specialist mental health care centers, regular general practitioners, and via self-referrals. Information about the trial was disseminated through flyers, social media, orientation meetings, and provided to general practitioners and local hospitals in the recruitment area. The information explicitly mentioned that participants had to have a history of depression and could not currently be in a major depressive episode (MDE). Potential participants were prescreened for exclusion criteria by telephone before in person clinical assessment and inclusion in study. Exclusion criteria were current or past neurological illness, bipolar disorder, psychosis, drug addiction and attention deficit disorder with and without hyperactivity (ADHD and ADD).

A sample of 120 participants from Oslo was included and recruited during the same time period as the 124 participants in the Sørlandet sample. The period of recruitment and follow-up was May 2015 to October 2018. All participants signed informed consents. The study was approved by the Norwegian Regional Committees for Medical and Health Research Ethics, reference number 2014/1989. To be eligible to participate in the study participants had to be between 18 and 65 years old and in remission with a history of MDD as established by the Mini International Neuropsychiatric Interview, version 6.0.0. (MINI) (Lecrubier and Sheehan, 1997).

### Procedure

Individuals referred for treatment (*N* = 273) were provided with initial information about the study by telephone prior to receiving a document containing further information and an informed consent form. Those who signed the consent form (*N* = 269) completed a baseline clinical assessment that included a study-specific questionnaire (where demographic information, including medication status, and treatment history were obtained), and administration of the MINI (Lecrubier and Sheehan, 1997). Psychologists or psychology students who had received training and supervision in the assessment package conducted the evaluations. Uncertainty regarding exclusion was discussed within a team of experienced psychologists. A total of 25 individuals were excluded or declined to participate in the study at this juncture.

The *Clinical Trials* protocol stated that current MDD was an exclusion criterion; however, 25 participants (10 participants from Oslo and 15 participants from Sørlandet) with ongoing MDD according to MINI criteria were included in error at baseline for a total sample of 244 participants. Because excluding data from these participants did not affect the results, they were included in all reported analyses. Tables presenting the analyses with these participants excluded can be found in the section “Supplementary Material.”

This multisite study consisted of two phases. In the first phase, 244 participants were randomized to either receive 14 days of ABM or a control condition without bias modification. This allocation was conducted by individuals not involved in the recruitment, assessment, treatment, or follow-up of participants using a 1:1 ratio randomization list that ensured that allocation was concealed from researchers and participants.

In a second quasi-experimental phase, the 124 participants included from Sørlandet were allocated to an 8-week, group-based ACT intervention. The 120 participants from Oslo received no specific intervention in phase 2 apart from regular health care provided by the Norwegian health services (i.e., outpatient treatment and/or treatment from general practitioners). Thus, the two phases of this study yielded a total of four groups: (a) Control + Control (no specific intervention), (b) ABM + Control (no specific intervention), (c) Control + ACT, and (d) ABM + ACT.

Outcome measures were administered and completed at baseline, 2 weeks (immediately after phase 1), as well as at 1, 2, 6, and 12 months after the conclusion of phase 1. In tables and figures, the follow-up will be shown as; 0 = baseline, 0.5 months = 2 weeks, 1.5 months, 2.5 months, 6.5 months, and 12.5 months.

**FIGURE 1:**
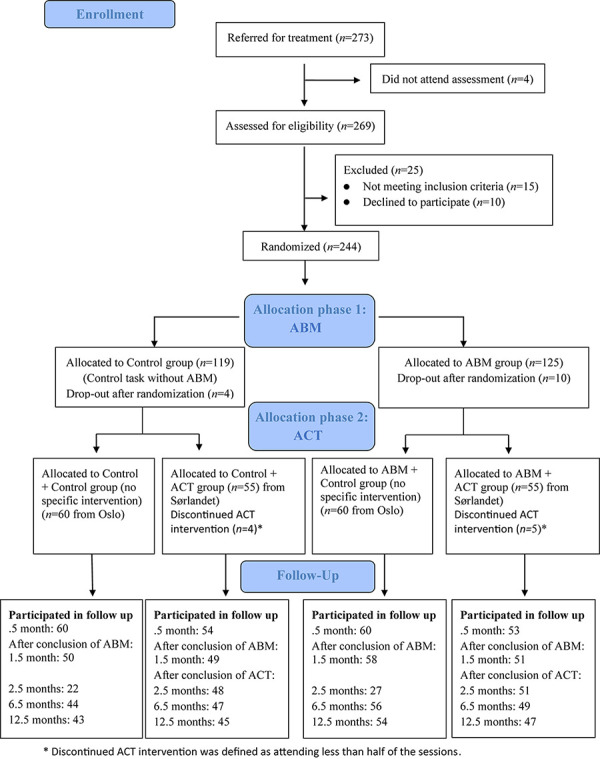
Flow chart of the study.

Figure 1 illustrates the flow of participants in the study. Data were available for 227 participants (93.4%) at 2 weeks, for 208 participants (85.2%) at 1 month, for 148 participants (60.6%) at 2 months, for 196 (80.3%) at 6 months, and for 189 participants (77.5%) at 12 months. See our *Clinical Trials* article (Østergaard et al., 2018) for power and sample size calculation.

### Outcome Measures

#### Primary Outcome

##### Beck depression inventory (BDI-II)

The Beck Depression Inventory II (BDI-II) (Beck et al., 1996) consists of 21 items, and is a psychometrically sound measure of depressive severity. The Norwegian translation of the BDI-II displays high internal consistency, and acceptable convergent and discriminative validity (Aasen, 2001). BDI-II was completed by participants at home and displayed excellent internal consistency at baseline (α = 0.92).

##### Hamilton rating scale (HRSD)

The Hamilton Rating Scale for Depression (HRSD) (Hamilton, 1960, 1967) is a widely used semistructured, clinical interview measuring the severity of a range of affective, behavioral, and biological symptoms of depression. The HRSD has acceptable psychometric properties (Rabkin and Klein, 1987) with good internal consistency (α = 0.79) and a high correlation (*r* = 0.58) with the BDI-II in this study at baseline.

#### Secondary Outcome

##### Recurrence of MDE

The MINI-International Neuropsychiatric Interview (MINI) (Lecrubier and Sheehan, 1997) is a structured diagnostic interview compatible with DSM-IV and ICD-10 criteria of assessing psychiatric diagnoses. The MINI have shown accuracy of depression in both psychiatric and primary care (Otsubo et al., 2005; de Azevedo Marques and Zuardi, 2008; Pettersson et al., 2018). Recurrence of MDE was assessed by the MINI version 6.0.0. at 12 months after phase 1.

### Feasibility Measure

To evaluate satisfaction and acceptability of the ACT group treatment, participants completed a brief survey about their experience upon its completion. Specifically, they responded to the following four statements according to a seven-point Likert scale: (a) “I’m satisfied with the ACT group treatment.” (b) “ACT group therapy has had a positive effect on my daily life.” (c) “I handle depressive symptoms better after having completed ACT group therapy,” and (d) “I would recommend ACT group treatment for a friend or family-member struggling with depression.”

## Interventions

### ABM

The computerized task as part of the ABM training was based on the dot probe procedure (Browning et al., 2012). In the current approach, participants initially focused on a fixation cross before being presented with two pictures of emotional faces (the stimuli) that could have the following valences: (a) positive, (b) neutral, or (c) negative (angry or fearful). After between 0.5 to 1 s one of the pictures was replaced by either one dot or two dots. Participants were instructed to press one of two buttons on the keyboard as quickly as possible to indicate the number of dots. A single training session consisted of 96 trials where the following combinations of valences were presented: (a) positive-neutral, (b) positive-negative, and (c) negative-neutral. In the ABM condition, dots appeared in the location of the positive stimulus 87% of the trials, encouraging attention toward such stimuli, and thus developing a more positive attentional bias. In the control condition, however, dots appeared 50% in both the positive and the negative stimuli. Participants received identical laptops with the task pre-installed, and were instructed to complete two training sessions every day at home over 14 days (a total of 28 sessions). A pre- and post-assessment of individual attentional biases was conducted by a single session of the control condition for both the ABM and control groups.

### ACT

Acceptance and commitment therapy as a group intervention for secondary prevention of depression was based on a detailed treatment manual in Norwegian developed for this study^1^. This protocol consisted of 8 sessions (meeting once a week for 2 1/2 h) with two therapists, and was based on the six processes that ostensibly promote and strengthen psychological flexibility, defined as “the ability to contact the present moment more fully as a conscious human being and to change, or persist in, behavior when doing so serves valued ends” (Hayes et al., 2006, p. 7). These processes are: (a) *acceptance* (developing an awareness and openness to private experiences), (b) *values* (identifying what is really important in life), (c) *defusion* (ability to observe thoughts as they are, from a detached perspective), (d) *committed action* (planning and performing actions that are guided by chosen values), (e) *self-as-context* (having our identity not defined by thoughts, emotions and experiences), and (f) *contact with the present moment* (focused and flexible attention in the here and now).

The treatment sessions combined psychoeducation with experiential exercises (e.g., getting in contact with a certain process by means of an illustrative activity) and group processing of both elements. Mindfulness exercises were performed at every session for participants to become present and observant of inner and outer experiences in the here-and-now. These exercises lasting from 5–15 min were provided in audio and were also assigned as daily homework. Additionally, participants were also assigned process-specific exercises at the end of each session to practice as homework. Except for the introduction (session 1) and summary/ending (session 8), each session targeted one of the six processes that are thought to contribute to psychological flexibility. The first four sessions, in particular, focused on increasing acceptance and awareness of difficult emotions and thoughts in order to shift attention away from controlling inner aversive experiences to what participants want their lives to be about. The last four sessions sought to integrate new perspectives and knowledge that can help participants clarify their goals and aspirations, and plan concrete actions that are in accordance with their values. See our study protocol (Østergaard et al., 2018) for a more detailed description about sessions.

### Therapists and Treatment Adherence

Acceptance and commitment therapy was administered by eight experienced psychologists who were trained in the approach and instructed to follow the manual which specified treatment ingredients, intervention structure and therapist behavior (Plumb and Vilardaga, 2010). Sessions were video-recorded with the consent of all the participants and a knowledgeable ACT researcher and clinician who was not involved in the treatment groups checked for adherence. Sessions were divided into 10 min modules that were chosen randomly for adherence checks. The manual identified which process to be assessed for adherence. Therapeutic stance and the occurrence and depth of ACT processes were evaluated. Based on the manual, the modules that were checked rated adherence level as either sufficient, or not sufficient. The majority (85%) was judged as having been conducted in a sufficient manner.

## Statistical Analyses

Statistical analyses were conducted using Stata/IC version 15.1 on an intention-to-treat basis. Growth curve modeling was used to determine whether the three treatment groups receiving either only ABM, only ACT, or a combination of ABM and ACT differed in outcome measures compared to the group assigned to control conditions within both phases of the study. Growth curve modeling allows investigation of within-persons variability in between-persons patterns of change over time, by treating participants‘ intercepts and slopes as random effects for estimation time (Curran et al., 2010). The growth model includes both fixed and random effects, making it possible to examine trajectory of change over time on both group (between-subjects) and individual levels (within-subjects). Because time is treated as a continuous rather than discrete variable, it has the potential of increasing statistical power in detecting the growth effects, while also allowing a flexible way of handling missing data (Kwok et al., 2008). Missing observation in data were not imputed, but handled by maximum likelihood estimations under the assumption of missing at random (MAR). The MAR assumption is common in clinical epidemiological research, and considered to be tenable (Donders et al., 2006).

A two-level growth model was built with level-1 modeling the repeated measures within participants and level-2 modeling the differences of individual growth models between participants (Kwok et al., 2007). Growth curve models with linear, quadratic, cubic, quartic, and quantic functions, as well as inclusion of different covariates were tested by using a likelihood-ratio test in order to find an acceptable model for the data (Chou et al., 2004). Maximum likelihood (ML) was used as the estimation method and an unstructured covariance structure was used in all models. To handle non-normally distributed data and heteroscedasticity in the residuals, we used a robust sandwich estimator to calculate standard errors. The statistical significance level was set to α = 0.05.

Multilevel effect sizes (ES) were calculated on several levels of the model. The global pseudo-*R*^2^ suggesting how much of the variance in outcome can be explained by the final model was estimated by squaring the correlation between observed and predicted outcome scores (Peugh, 2010). ES for the coefficients of primary interest; i.e., the cross-level coefficients between time and treatment were calculated by using the formula B_11_ (time)/SD_*RAW*_ suggested by Feingold (2009).

## Results

### Participants

There were no significant differences between the ABM and control groups in gender, age, educational level, antidepressant medication status, comorbidity, and depressive symptoms at baseline (phase 1). There, however, were significant differences between the control and ACT groups at phase 2 in educational level, comorbidity, and in BDI-II scores at baseline. These differences were taken into account in the growth curve model by individually adjusting the lines of regression. Table 1 shows sample characteristics.

**TABLE 1:** Sample characteristics at baseline, given as number and proportion for categorical characteristics and as mean and standard deviation for quantitative characteristics.

	**Phase 1**			**Phase 2**	
**Characteristics**	**Control *n* = 119**	**ABM *n* = 125**	**Control + Control *n* = 60**	**ABM + Control *n* = 60**	**Control + ACT *n* = 59**	**ABM + ACT *n* = 65**
Gender						
Males	32 (26.9)	34 (27.2)	17 (28.3)	16 (26.7)	15 (25.4)	18 (27.7)
Females	87 (73.1)	91 (72.8)	43 (71.7)	44 (73.3)	44 (74.6)	47 (72.3)
Age	38.0 (12.7)	38.8 (12.4)	36.5 (13.1)	35.3 (12.2)	39.6 (12.2)	42.0 (11.8)
Education						
Lower than	34 (28.6)	30 (24.0)	9 (15.0)	8 (13.3)	25 (42.4)	22 (33.8)
university	78 (65.5)	87 (69.6)	45 (75.0)	46 (76.7)	33 (55.9)	41 (63.1)
University or higher	7 (5.9)	8 (6.4)	6 (10.0)	6 (10.0)	1 (1.7)	2 (3.9)
Missing						
Antidepressants						
No	81 (68.1)	85 (68.0)	41 (68.3)	45 (75.0)	40 (67.8)	40 (61.5)
Yes	35 (29.4)	36 (28.8)	(31.7)19	15 (25.0)	16 (27.1)	21 (32.3)
Missing	3 (2.5)	4 (3.2)	−	−	3 (5.1)	4 (6.2)
Comorbidity No	47 (39.5)	53 (42.4)	21 (35.0)	15 (25.0)	26 (44.1)	38 (58.5)
Yes	72 (60.5)	72 (57.6)	39 (65.0)	45 (75.0)	33 (55.9)	27 (41.5)
Episodes depression	5.4 (6.7)	6.1 (8.6)	4.8 (6.3)	4.8 (7.9)	5.9 (7.1)	7.2 (9.1)
Missing	1 (0.1)	1 (0.1)	−	1 (1.7)	1 (1.7)	−
BDI-II score	16.9(10.9)	19.0 (19.5)	12.8 (8.3)	16.0 (11.0)	21.5 (11.7)	22.3 (9.0)
Missing	5 (4.2)	10 (8.0)	−	−	5 (8.5)	10 (15.4)
HRSD score	9.5 (5.6)	9.4 (6.2)	8.4 (4.7)	9.7 (6.4)	10.7 (6.3)	9.2 (6.0)
Missing	3 (2.5)	−	−	−	3 (5.1)	−

### Feasibility

Of the 124 participants allocated to the group-based ACT treatment, 14 never attended treatment and 9 discontinued it (defined as attending less than half of the sessions). On average, the remaining participants attended 6.75 of the 8 sessions (*n* = 100, range 4–8 sessions). At the end of treatment, the following mean scores were found for satisfaction with it (*n* = 86, Scale 1–7): (a) “I’m satisfied with the group-based ACT treatment” (*M* = 6.17, *SD* = 0.08), (b) “ACT group therapy has had a positive effect on my daily life” (*M* = 5.73, *SD* = 1.19), (c) “I handle depressive symptoms better after having completed group-based ACT treatment” (*M* = 5.52, *SD* = 1.20), and (d) “I would recommend group-based ACT treatment for a friend or family-member struggling with depression” (*M* = 6.29, *SD* = 1.24).

### Outcomes

#### Primary Outcomes

In the growth curve model, we first sought to investigate whether BDI-II and HRSD scores decreased as a function of time, and if these variables changed differently for the two groups receiving ACT compared to the two groups receiving no specific intervention in phase 2. Model based means and standard deviations during follow-up assessments are presented in Table 2. The results from the growth curve model, that scales time in months, are presented in Table 3 (BDI-II) and Table 4 (HRSD).

**TABLE 2:** Model based mean value and standard deviation during follow-up separated on treatment.

	**Control + Control**	**ABM + Control**	**Control + ACT**	**ABM + ACT**
**Months**	**Mean (SD)**	**Mean (SD)**	**Mean (SD)**	**Mean (SD)**
*BDI-II*				
0	12.02(6.62)	14.71(9.44)	21.48(9.17)	21.93(7.26)
0.5	10.96(6.35)	13.71(9.10)	19.72(8.75)	20.26(7.45)
1.5	9.41(6.42)	12.25(9.01)	16.93(8.83)	17.61(8.34)
2.5	8.54(6.67)	11.44(9.17)	14.98(9.20)	15.77(9.11)
6.5	9.72(6.24)	12.46(8.80)	13.00(8.34)	13.96(8.63)
12.5	13.79(8.27)	14.65(10.60)	12.89(8.82)	13.65(8.64)
*HRSD*				
0	8.43(3.26)	9.25(3.93)	10.11(4.05)	9.20(3.95)
0.5	8.19(3.26)	9.01(3.95)	9.64(4.03)	8.79(3.94)
1.5	7.79(3.25)	8.66(3.99)	8.77(4.00)	8.05(3.93)
2.5	7.48(3.26)	8.38(4.03)	7.79(3.98)	7.40(3.92)
6.5	7.13(3.34)	8.17(4.28)	5.76(3.91)	5.68(3.95)
12.5	9.29(3.65)	10.55(4.79)	5.11(3.93)	5.81(4.13)

**TABLE 3:** Growth curve model for estimates of Beck’s Depression Inventory scale, comparing three treatment groups vs. controls.

**Parameters**	**Unconditional**	**Level 1**	**Level 2**	**Cross level interaction**
**Fixed effects**				
Intercept	14.92^(0.61)***^	17.39^(0.72)***^	12.31^(1.74)***^	11.01^(1.78)***^
Time				
Months, linear		−2.60^(0.51)***^	−2.86^(0.52)***^	−2.54^(0.53)***^
Months, quadratic		0.39^(0.11)***^	0.44^(0.11)***^	0.46^(0.11)***^
Months, cubic		−0.02^(0.01)**^	−0.02^(0.01)**^	−0.02^(0.01)**^
Treatment				
ABM + Control			2.32(1.55)^n.s.^	2.52(1.60)^n.s.^
Control + ACT			6.15^(1.59)***^	8.82^(1.75)***^
ABM + ACT			7.37^(1.42)***^	9.98^(1.51)***^
Higher education			−3.25^(1.45)*^	−3.27^(1.43)*^
Antidepressant treatment			3.41^(1.38)*^	3.31^(1.37)*^
Comorbidity			4.39^(1.03)***^	4.45^(1.01)***^
Interaction				
(ABM + Control) × Months, linear				−0.12(0.18)^n.s.^
(Control + ACT) × Months, linear				−0.80^(0.17)***^
(ABM + ACT) × Months, linear				−0.77^(0.18)***^
**Random effects**				
sd (Residuals)	7.26 (0.32)	5.18 (0.30)	5.14 (0.31)	5.13 (0.31)
sd (Intercept)	8.53 (0.57)	9.91 (0.60)	8.50 (0.59)	8.35 (0.57)
sd (Months, linear)		4.99 (0.61)	5.05 (0.63)	5.03 (0.63)
sd (Months, quadratic)		0.96 (0.14)	0.97 (0.14)	0.98 (0.14)
sd (Months, cubic)		0.05 (0.01)	0.05 (0.01)	0.05 (0.01)
Correlation (Months, linear; Intercept)		−0.37(0.11)	−0.38(0.10)	−0.36(0.10)
Correlation (Months, linear; Months quadratic)		−0.97(0.01)	−0.97(0.01)	−0.97(0.01)
Correlation (Months, linear; Months cubic)		0.92 (0.03)	0.92 (0.03)	0.92 (0.03)
Correlation (Months, quadratic; Intercept)		0.30 (0.11)	0.32 (0.11)	0.30 (0.11)
Correlation (Months quadratic, Months cubic)		−0.99(0.01)	−0.99(0.01)	−0.99(0.01)
Correlation (Months, cubic; Intercept)		−0.28(0.11)	−0.29(0.11)	−0.29(0.11)
**Model summary**				
Deviance statistic	8,470.86	8,283.85	7,661.69	7,587.61
Number of estimated parameters	3	15	21	24

*Robust standard errors in parentheses. n.s. – non-significant. ^∗^*p* < 0.05. ^∗∗^*p* < 0.01. ^∗∗∗^*p* < 0.001.*

**TABLE 4:** Growth curve model for estimates of the Hamilton Depression Rating Scale, comparing three treatment groups vs. controls.

**Parameters**	**Unconditional**	**Level 1**	**Level 2**	**Cross level interaction**
**Fixed effects**				
Intercept	8.30^(0.30)***^	9.22^(0.33)***^	6.59^(0.67)***^	6.12^(0.69)***^
Time				
Months, linear		−0.64^(0.11)***^	−0.67^(0.11)***^	−0.49^(0.12)***^
Months, quadratic		0.04^(0.01)***^	0.04^(0.01)***^	0.04^(0.01)***^
Treatment				
ABM + Control			0.87(0.76)^n.s.^	0.68(0.78)^n.s.^
Control + ACT			0.70(0.80)^n.s.^	1.95^(0.86)*^
ABM + ACT			0.46(0.77)^n.s.^	1.35(0.81)^n.s.^
Antidepressant treatment			1.87^(0.69)**^	1.82^(0.69)**^
Comorbidity			2.63^(0.55)***^	2.66^(0.55)***^
Interaction				
(ABM + Control) × Months, linear				0.03(0.10)^n.s.^
(Control + ACT) × Months, linear				−0.47^(0.10)***^
(ABM + ACT) × Months, linear				−0.34^(0.10)**^
**Random effects**				
sd (Residuals)	4.32 (0.16)	3.92 (0.15)	3.90 (0.16)	3.90 (0.16)
sd (Intercept)	4.20 (0.28)	4.19 (0.29)	3.95 (0.29)	3.92 (0.28)
sd (Months, linear)		0.34 (0.04)	0.34 (0.04)	0.27 (0.05)
Correlation (Months, linear; Intercept)		−0.09(0.11)	−0.16(0.13)	−0.18(0.12)
**Model summary**				
Deviance statistic	7,256.92	7,182.11	6,972.69	6,933.42
Number of estimated parameters	3	7	12	15

*Robust standard errors in parentheses, n.s. – non-significant. ^∗^*p* < 0.05. ^∗∗^*p* < 0.01. ^∗∗∗^*p* < 0.001.*

**FIGURE 2:**
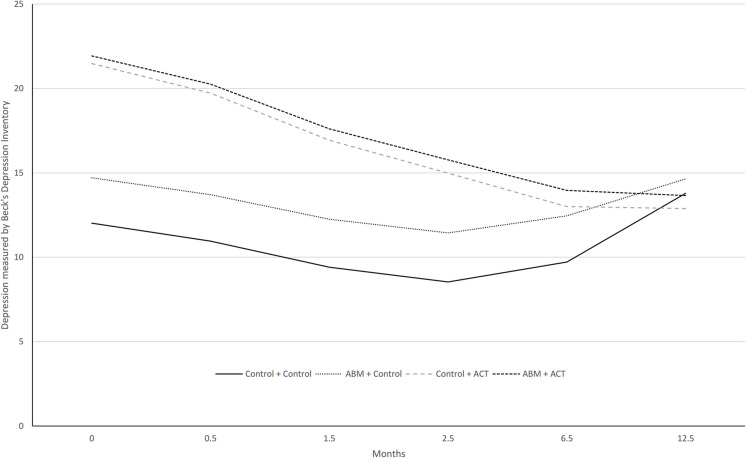
Model-based trajectories on BDI over 12 months follow-up.

Figure 2 shows the course of improvement in self-reported levels of depression as assessed by the BDI-II over 12 months. The global pseudo *R*^2^ ES for the cross-level interaction model was [(0.426)^2^ × 100] = 18.15; i.e., 18% of the variation in self-reported depression scores can be explained by the model. The two ACT groups exhibited a significant reduction per month in BDI-II scores over a year compared to the two groups who did not receive this treatment during phase 2. The control + ACT group had a monthly reduction in self-reported depression of −0.80 points (ES = –1.21), and the ABM + ACT group a reduction of −0.77 points (ES = –1.16). The ABM + control group exhibited a non-significant reduction in BDI-II scores of −0.12 points (ES = −0.18).

**FIGURE 3:**
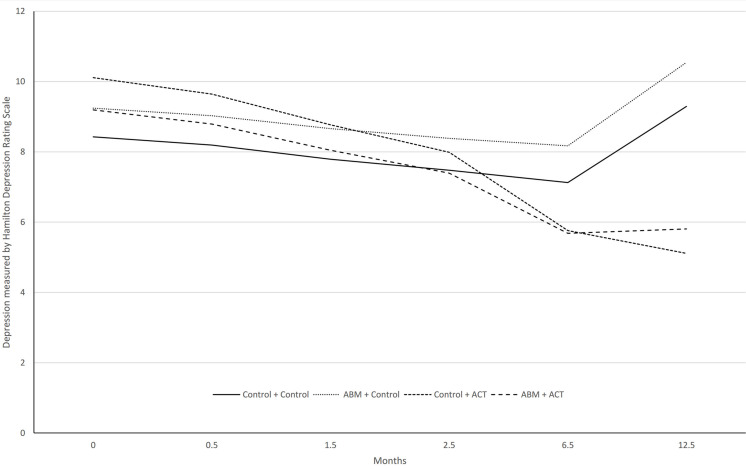
Model-based trajectories on HRSD over 12-month follow-up.

Figure 3 shows the course of improvement in levels of clinician-rated depression over 12 months. The global pseudo *R*^2^ ES for the cross-level interaction model was [(0.346)^2^ × 100] = 11.97, i.e., 12% of the variation in clinician-rated depression scores can be explained by the model. The two ACT groups also exhibited a significant reduction per month in HRSD scores over a year compared to the two groups who did not receive this treatment during phase 2. The Control + ACT group had a monthly reduction in HRSD scores of -0.47 points (ES = –1.25) and the ABM + ACT group a reduction of -0.34 points (ES = –0.91). By contrast, there was a non-significant monthly increase of 0.03 points (ES = 0.08) in the ABM + Control group.

#### Secondary Outcome

At the 12-months follow-up, using the criteria of the MINI structured interview, we found that 21% of participants (*n* = 47) in the ABM + ACT group, 21% (*n* = 43) in the control + ACT group, 38% (*n* = 50) in the ABM + control group, and 53% (*n* = 40) in the control + control group had experienced at least one MDE during the previous year. These proportions were significantly lower in two ACT groups than in the two other conditions (χ^2^_(__1__)_ = 11.12, *p* < 0.001).

## Discussion

The present study investigated the impact of ACT as a stand-alone intervention for individuals with a history of depression, and whether preceding it with ABM improves outcome. To our knowledge this is the first study investigating ACT and relapse prevention of MDD, and the combination of ABM and a specific therapy in treatment of depression.

At 1-year follow-up, the two groups receiving ACT showed a continuing and significant reduction of large ES in both self-reported and clinician-rated measures of depressive symptoms. These results are consistent with earlier studies that found ACT interventions to be effective in remitting depressive symptoms (Twohig and Levin, 2017), and also confirm the effectiveness of ACT for depression in a group-format (Zettle and Rains, 1989). By contrast, over this same period of time, the other two conditions that did not receive ACT in phase 2, showed relapse back to baseline levels of depression on both scales. Insofar as residual symptoms of depression are a central risk factor in recurrence of MDD (Cuijpers and Smit, 2004), our findings suggest that group-based ACT could serve a preventive function. This tentative assessment is also supported by the significantly lower recurrence rate of MDEs over the course of this project among those receiving ACT.

The absence of any significant difference between the two conditions that received ACT in their monthly reduction in depressive symptoms during treatment or at follow-up suggests that ABM, contrary to expectations, did not further contribute to the therapeutic impact of ACT. To our knowledge there has only been one previous study that combined ABM and psychotherapy in the treatment of depression. Vrijsen et al. (2018) investigated the combination of two different versions of ABM and treatment-as-usual (TAU) in clinical depression. The 14 days of treatment took place in an inpatient setting where TAU was defined as any clinical treatment offered there. Unlike the two distinct phases of this study, two versions of ABM training (cognitive bias modification and attention dot-probe training) were introduced *while* the patients were receiving TAU. Both types of ABM contributed to a significant decrease in self-reported depressive symptoms compared to TAU without either type of ABM training, but not on clinician-rated measures.

There may be several reasons why we found no boost for ABM in our study. First, the results reveal that group-based ACT treatment had a strong effect in reducing depressive symptoms, which may have contributed to a floor effect in which any additive impact of ABM was difficult to detect. It may also be that breaking the effects down to single symptoms and their interactions might have shown some add on effects; see Kraft et al. (2019).

Our study followed a different procedure than Vrijsen et al. (2018) by introducing ABM *before* ACT treatment started. It could be that ABM training would have significantly contributed to the impact of ACT if it had been provided concurrent with it. ABM and ACT both ostensibly seek to increase ability to shift attentional focus, but in quite differing ways. ABM addresses attention through implicit processes (Jones and Sharpe, 2017), while ACT explicitly targets rule-governed behavior by bringing certain facets of present moment awareness under deliberate control. To benefit from the combination of ABM and ACT it could be argued that the different processes need to be activated simultaneously. However, this would have affected the feasibility of the treatment, and could have appreciably added to participant treatment burden to where drop-out rates increased. Furthermore, it may be that ABM training needs to unfold over a longer period of time in order to have a carry-over effect to other treatments that it precedes.

Another consideration in evaluating the impact or lack thereof for ABM in this study involves variations in such training. The ABM task applied in this study was more static and less complex than other existing training procedures that incorporate more complex displays and multiple inputs of emotionally relevant stimuli (e.g., emotion-in-motion and visual search task) (Van Bockstaele et al., 2019). It could be that ABM training that is more engaging and task-oriented, in which participants are more oriented toward and instructed to find emotionally relevant stimuli, would be more beneficial when combined with psychotherapy (Jones and Sharpe, 2017). Furthermore, the dot-probe task has been criticized for lack of sensitivity in tracking attention during the execution of the task (LeMoult and Gotlib, 2018). Recent studies (Vazquez et al., 2016; Beevers et al., 2019) have suggested that eye-tracking technology could overcome this limitation, allowing a continuous monitoring of visual attention. Future studies may want to adopt this technology in order to increase precision of attention tracking.

A final factor that may have contributed to the weak effect of ABM training in this study was that it was done at home, which might have affected the compliance and execution of the training. A recent meta-analysis (Jones and Sharpe, 2017) concluded that laboratory-based training is more effective than home-based training. This might especially be the case when combining treatments in order not to overwhelm participants with homework assignments.

We would be remiss to not acknowledge several limitations in this study. First, the randomization was suboptimal in the sense that no participants from Oslo received ACT in phase 2. Moreover, the participants from Sørlandet during phase 1 knew that they would receive additional treatment, which might have impacted the results. Second, phase 2 of the study was not a randomized design, but a quasi-experimental one. Although this design is more pragmatic in nature, it decreases internal validity (Patsopoulos, 2011). Third, we did not monitor what kind of intervention the control group received in the follow-up period, and if the participants that received group-based ACT also had additional treatment that might have had an effect on the outcome. Fourth, in order to reduce participant burden in the follow-up assessments, we only determined recurrence of MDEs by the MINI structured interview at 12 months. Ideally, this interview should have been administered at all measurement occasions to consider rates of relapse in participants reliably and how they unfold over time. Fifth, we deviated from the preregistered protocol and included a small number of participants who also fulfilled the formal criteria for current MDD. Even though the large majority of participants did not meet the MINI criteria for major depression, the mean level of baseline depression as assessed by BDI-II scores fell within the 12.8–22.3 range and was larger in participants from Sørlandet. A possible explanation for this discrepancy in baseline BDI-II scores is that a substantial number of participants from Sørlandet were recruited to this study after initially having been rejected treatment from an outpatient clinic (because they did not meet criteria of a MDE). This might have contributed to an increased subjective experience of depressive symptoms in these participants that did not become expressed in the same way in the HRSD scores. Sixth, there were a substantial number of missing data at 2.5 months at the Oslo site because of challenges in follow-up at that juncture. Although this represents a potential bias, the strength of the growth curve model performed in this study is its ability to handle missing data. There were no logistic or recruitment differences between sites.

A strength of the study was that including a heterogeneous group of participants with regards to their intensity of residual symptoms, previous depressive episodes, and concurrent comorbidity, did not limit the effectiveness of treatment. Also, we did not include a waiting list control group that have been found to overestimate treatment effects (Cunningham et al., 2013). The participants were unselected patients in a standard outpatient clinic, thus representing the general population of those seeking mental health services. Collectively, this suggests that the findings are generalizable (Maciejewski et al., 2013). A further strength of the study involved tracking levels of depression with both clinician-rated and self-reported measurements of depression over a lengthy follow-up period (12 months). The outcome measures are both well-validated. Advanced statistical analysis was conducted in order to increase precision of estimates in the presence of missing data and differences between sites.

In conclusion, our findings suggest that ACT is an effective treatment for residual symptoms of depression in a “real-world” clinical setting, where patients have a history of depression and present themselves with varied histories, backgrounds, and challenges. Receiving group-based ACT treatment significantly reduced depressive symptomatology on both clinician-rated and self-report scales that were maintained and continued over the course of a 1-year follow up. This suggests that ACT successfully sets in motion processes that could prevent relapse. The results in the current study did not support our expectation that combining ABM and ACT would augment the latter in a way that increases reduction in residual symptoms. There are several approaches to ABM and different ways to administer the training (Cristea et al., 2015; Jones and Sharpe, 2017). Future research should explore if some types and perhaps “dosages” of ABM tasks fit better with mechanisms of specific treatments, and if combining ABM and treatment approaches such as ACT more directly, could induce potential complementary mechanisms of therapeutic change.

## Data Availability

The datasets generated for this study are available on request to the corresponding author.

## Ethics Statement

Human Subject Research: The studies involving human participants were reviewed and approved by the Norwegian Regional Committees for Medical and Health Research Ethics, reference number 2014/1989. The participants provided their written informed consent to participate in this study.

## Author Contributions

TØ contributed to the study conception and design, project planning, acquisition, analysis and interpretation of the data, and drafted the manuscript. TL and CH contributed to the study design, project planning, and critical revision. IR contributed to the analysis and interpretation of the data, and critical revision. RZ contributed to the study conception and design, and critical revision. RJ contributed to the study conception and design, project planning, interpretation of the data, and critical revision. TS and NL contributed to the study conception and design, project planning, and critical revision. VH contributed to the study design, project planning, analysis and interpretation of the data, and critical revision. All authors read and approved the final manuscript.

## Conflict of Interest Statement

CH has received consultancy fees from Johnson & Johnson Inc., P1vital, and Lundbeck. NL has received consultancy fees and travel expenses from Lundbeck. The remaining authors declare that the research was conducted in the absence of any commercial or financial relationships that could be construed as a potential conflict of interest.

## Footnotes

1 To receive a copy of the manual please contact the first author.
