# Corrigendum: Acceptance and commitment therapy preceded by attention bias modification on residual symptoms in depression: a 12-month follow-up

**DOI:** 10.3389/fpsyg.2023.1233621

**Published:** 2023-08-03

**Authors:** Tom Østergaard, Tobias Lundgren, Ingvar Rosendahl, Robert D. Zettle, Rune Jonassen, Catherine J. Harmer, Tore C. Stiles, Nils Inge Landrø, Vegard Øksendal Haaland

**Affiliations:** ^1^Department of Psychiatry, Sørlandet Hospital, Arendal, Norway; ^2^Department of Psychology, Clinical Neuroscience Research Group, University of Oslo, Oslo, Norway; ^3^Department of Clinical Neuroscience, Center for Psychiatry Research, Karolinska Institutet and Stockholm Health Care Services, Stockholm County Council, Stockholm, Sweden; ^4^Department of Psychology, Wichita State University, Wichita, KS, United States; ^5^Psychopharmacology and Emotion Research Laboratory, University Department of Psychiatry, Oxford, United Kingdom; ^6^Department of Psychology, Norwegian University of Science and Technology, Trondheim, Norway; ^7^Division of Psychiatry, Diakonhjemmet Hospital, Oslo, Norway

**Keywords:** acceptance and commitment therapy, attentional bias modification, combined treatment, depression, residual symptoms

In the published article, there was an error in the Acknowledgments section. The Coperio Centre was mentioned as an external recruitment site. However, no participants were recruited from the Coperio Centre. The corrected Acknowledgments section appears below:

