# Supplementary material for: Acceptance and Commitment Therapy Preceded by Attention Bias Modification on Residual Symptoms in Depression: A 12-Month Follow-Up
Source: Front Psychol. 2019 Aug 29;10:1995. doi: 10.3389/fpsyg.2019.01995 (PMC6727662; doi:10.3389/fpsyg.2019.01995)
Supplement: Supplementary file 1 [file Table_1.DOCX]

Supplementary Table 1. Growth curve model for estimates of Beck’s Depression Inventory scale, comparing three treatment groups vs. controls with 25 participants included in error dropped.

| Parameters | Unconditional | Level 1 | Level 2 | Cross level interaction |
| --- | --- | --- | --- | --- |
| *Fixed effects* |  |  |  |  |
| Intercept | 13.65 (.57)*** | 15.96 (.72)*** | 12.66 (1.60)*** | 11.28 (1.61)*** |
| Time |  |  |  |  |
| Months, linear |  | -2.54 (.53)*** | -2.88 (.55)*** | -2.54 (.55)*** |
| Months, quadratic |  | .39 (.11)*** | .45 (.11)*** | .47 (.11)*** |
| Months, cubic |  | -.02 (.01)** | -.02 (.01)** | -.02 (.01)** |
| Treatment |  |  |  |  |
| ABM + Control |  |  | 1.50 (1.39)^n.s.^ | 1.80 (1.46)^n.s.^ |
| Control + ACT |  |  | 4.98 (1.59)*** | 8.12 (1.72)*** |
| ABM + ACT |  |  | 5.92 (1.34)*** | 8.84 (1.43)*** |
| Higher education |  |  | -2.42 (1.34)* | -2.54 (1.32)^n.s.^ |
| Antidepressant treatment |  |  | .88 (1.16)^n.s.^ | .80 (1.15)^n.s.^ |
| Comorbidity |  |  | 3.44 (.98)*** | 3.50 (.97)*** |
| Interaction |  |  |  |  |
| (ABM + Control) * Months, linear |  |  |  | -.13 (.19)^n.s.^ |
| (Control + ACT) * Months, linear |  |  |  | -.88 (.18)*** |
| (ABM + ACT) * Months, linear |  |  |  | -.80 (.19)*** |
| *Random effects* |  |  |  |  |
| sd (Residuals) | 7.17 (.35) | 5.18 (.20) | 5.12 (.33) | 5.11 (.33) |
| sd (Intercept) | 7.34 (.50) | 9.26 (.57) | 8.11 (.72) | 7.82 (.63) |
| sd (Months, linear) |  | 4.83 (.57) | 4.89 (.68) | 4.79 (.68) |
| sd (Months, quadratic) |  | .88 (.14) | .88 (.16) | .88 (.16) |
| sd (Months, cubic) |  | .05 (.01) | .04 (.01) | .05 (.01) |
| Correlation (Months, linear; Intercept) |  | -.50 (.10) | -.52(.11) | -.48 (.12) |
| Correlation (Months, linear; Months quadratic) |  | -.97 (.01) | -.92 (.03) | -.98 (.03) |
| Correlation (Months, linear; Months cubic) |  | .92 (.03) | .92 (.03) | .93 (.03) |
| Correlation (Months, quadratic; Intercept) |  | .44 (.11) | .46 (.12) | .42 (.13) |
| Correlation (Months quadratic, Months cubic) |  | -.99 (.01) | -.99 (.01) | -.99 (.01) |
| Correlation (Months, cubic; Intercept) |  | -.42 (.12) | -.43 (.13) | -.39 (.13) |
| *Model summary* |  |  |  |  |
| Deviance statistic | 7,405.36 | 7,245.91 | 6,667.90 | 6,634.46 |
| Number of estimated parameters | 3 | 15 | 21 | 24 |

Note: Robust standard errors in parentheses

n.s. = non significant

* *p* < .05

** *p* < .01

*** *p* < .001
