# Supplementary material for: Acceptance and Commitment Therapy Preceded by Attention Bias Modification on Residual Symptoms in Depression: A 12-Month Follow-Up
Source: Front Psychol. 2019 Aug 29;10:1995. doi: 10.3389/fpsyg.2019.01995 (PMC6727662; doi:10.3389/fpsyg.2019.01995)
Supplement: Supplementary file 2 [file Table_2.docx]

Supplementary Table 2. Growth curve model for estimates of the Hamilton Depression Rating Scale, comparing three treatment groups vs. controls with 25 participants included in error dropped.

| Parameters | Unconditional | Level 1 | Level 2 | Cross level interaction |
| --- | --- | --- | --- | --- |
| *Fixed effects* |  |  |  |  |
| Intercept | 7.76 (.29)*** | 8.48 (.32)*** | 6.71 (.62)*** | 6.35 (.64)*** |
| Time |  |  |  |  |
| Months, linear |  | -.53 (.11)*** | -.56 (.11)*** | -.41 (.13)** |
| Months, quadratic |  | .04 (.01)*** | .04 (.01)*** | .04 (.01)*** |
| Treatment |  |  |  |  |
| ABM + Control |  |  | .34 (.74)^n.s.^ | .04 (.76)^n.s.^ |
| Control + ACT |  |  | .42 (.76)^n.s.^ | 1.61 (.83)^n.s.^ |
| ABM + ACT |  |  | -.25 (.76)^n.s.^ | .47 (.79)^n.s.^ |
| Antidepressant treatment |  |  | .97 (.65)^n.s.^ | 1.82 (.69)^n.s.^ |
| Comorbidity |  |  | 2.34 (.54)*** | 2.37 (.54)*** |
| Interaction |  |  |  |  |
| (ABM + Control) * Months, linear |  |  |  | .08 (.10)^n.s.^ |
| (Control + ACT) * Months, linear |  |  |  | -.48 (.10)*** |
| (ABM + ACT) * Months, linear |  |  |  | -.30 (.10)** |
| *Random effects* |  |  |  |  |
| sd (Residuals) | 3.79 (.24) | 3.73 (.15) | 3.70 (.16) | 3.69 (.15) |
| sd (Intercept) | 4.12 (.10) | 3.71 (.23) | 3.55 (.25) | 3.51 (.23) |
| sd (Months, linear) |  | .34 (.04) | .35 (.04) | .27 (.05) |
| Correlation (Months, linear; Intercept) |  | -.06 (.14) | -.19 (.17) | -.18 (.12) |
| *Model summary* |  |  |  |  |
| Deviance statistic | 6,297.33 | 6,232.89 | 6,031.18 | 5,991.50 |
| Number of estimated parameters | 3 | 7 | 12 | 15 |

Note: Robust standard errors in parentheses

n.s. = non significant

* *p* < .05

** *p* < .01

*** *p* < .001
